# Whole-transcriptome analysis of Aortic Stenosis reveals dysregulated RNA networks, immune cell infiltration, and NADK2 as a candidate regulator

**DOI:** 10.1186/s41065-026-00675-w

**Published:** 2026-04-17

**Authors:** Feng-xia Wang, Ming-jun Duan, Hao-qiang Guo, Jia-qing Yu, Fen Liu, Nilupaer Aisikeer, Yi-tong Ma, Xiang Xie

**Affiliations:** 1 Department of Cardiology, First Affiliated Hospital of Xinjiang Medical University, No. 137, Liyushan South Road, Xincheng District, Urumqi, Xinjiang Uygur Autonomous Region 830000 China; 2 Animal Experimental Center, Xinjiang Medical University, Urumqi, Xinjiang Uygur Autonomous Region 830017 China; 3 Department of Human Anatomy, School of Basic Medical Sciences, Xinjiang Medical university, Urumqi, Xinjiang Uygur Autonomous Region 830017 China; 4 Xinjiang Key Laboratory of Cardiovascular Disease Research, Urumqi , Xinjiang Uygur Autonomous Region 830000 China

**Keywords:** Aortic stenosis, Immune infiltration, NADK2, Transcriptomics

## Abstract

**Background:**

Aortic stenosis (AS) is a common cardiovascular condition marked by progressive narrowing of the aortic valve and is associated with substantial morbidity and mortality. Although its clinical impact is well recognized, the molecular mechanisms driving AS progression remain incompletely defined, particularly with regard to the contributions of non-coding RNAs and immune–metabolic interactions.

**Methods:**

High-throughput RNA sequencing combined with integrative bioinformatics analyses was performed on human aortic valve tissues from patients with AS to identify differentially expressed RNAs. Expression of Nicotinamide Adenine Dinucleotide Kinase 2 (NADK2) was validated using western blotting and quantitative polymerase chain reaction in both human AS specimens and murine AS models. Functional enrichment analyses, immune cell infiltration profiling, and weighted gene co-expression network analysis were applied to characterize regulatory networks and identify hub genes.

**Results:**

A total of 1,443 messenger RNAs (mRNAs), 3,147 long non-coding RNAs (lncRNAs), and 145 circular RNAs (circRNAs) were differentially expressed in AS valve tissues, with the majority showing increased expression. Principal component analysis demonstrated clear separation between AS and control samples. Functional enrichment analyses linked differentially expressed mRNAs primarily to sensory perception and calcium signaling pathways, lncRNAs to embryonic development–related processes, and circRNAs to protein regulatory functions. NADK2 was identified as a hub gene and was significantly upregulated at both the mRNA and protein levels in valve tissues from patients with AS and in the murine AS model. Immune infiltration analysis indicated increased proportions of CD4⁺ memory T cells and CD8⁺ T cells in AS, with strong positive correlations observed between these immune cell populations and NADK2 expression. Weighted gene co-expression network analysis further supported NADK2 as a key candidate regulator within the disease-associated magenta module.

**Conclusion:**

This study delineates comprehensive RNA regulatory networks in patients with AS and identifies NADK2 as a key molecular contributor to disease pathogenesis. Its consistent upregulation across species, together with close associations with immune dysregulation and co-expression network modules, supports its potential relevance as a therapeutic target in AS.

**Supplementary Information:**

The online version contains supplementary material available at 10.1186/s41065-026-00675-w.

## Introduction

Aortic stenosis (AS) is a common cardiovascular disease characterized by progressive narrowing of the aortic valve, leading to impaired cardiac function and adverse clinical outcomes [1]. Its clinical significance is underscored by a rising prevalence in the general population and the substantial burden it places on healthcare systems worldwide [2]. Although multiple molecular and cellular mechanisms have been implicated in AS pathogenesis, important gaps remain in the understanding of differential RNA expression and its functional relevance in this condition [3].

Genetic factors contribute to both the development and progression of AS [4]. However, systematic characterization of messenger RNAs (mRNAs), long non-coding RNAs (lncRNAs), and circular RNAs (circRNAs) within this disease context remains limited. These RNA species play essential roles in regulating gene expression and cellular processes, and their coordinated interactions may offer important insights into the molecular basis of AS [5, 6]. Clarifying these regulatory relationships may facilitate the identification of novel biomarkers and therapeutic targets with potential relevance to clinical outcomes.

In this study, high-throughput RNA sequencing combined with advanced bioinformatics analyses was employed to characterize the expression profiles of mRNAs, lncRNAs, and circRNAs in patients with AS. The objectives were to define the biological significance of differentially expressed RNAs, examine their associations with immune cell infiltration, and assess their relationships with metabolic pathways relevant to AS. Integration of high-throughput sequencing with bioinformatics represents a well-established approach in molecular biology, enabling comprehensive investigation of complex biological processes such as those underlying AS [7, 8]. Through analysis of the diverse contributions of multiple RNA species, this work sought to address existing knowledge gaps and to establish a foundation for future research focused on targeted therapeutic development.

In summary, the transcriptomic landscape of AS was examined through RNA expression profiling, providing insights into potential biomarkers and therapeutic targets. These findings extend beyond mechanistic understanding, as they may also inform clinical practice and support improved management strategies for patients with AS. Continued investigation into the interactions among RNA species may further advance understanding of AS pathophysiology and help identify new opportunities for therapeutic intervention.

## Methods

### Study population

This study included patients with AS who were hospitalized at the People’s Hospital of Xinjiang Uygur Autonomous Region. The AS group (*n* = 8) comprised patients diagnosed with severe calcific aortic valve disease (CAVD) who underwent surgical aortic valve replacement (SAVR). The control group (*n* = 8) consisted of age-matched patients undergoing surgical treatment for non-calcific aortic root pathologies, including ruptured sinus of Valsalva aneurysm, Stanford type A aortic dissection, or aortic root aneurysmal dilation.

For inclusion in the control group, aortic valve leaflets were required to be free of stenosis and significant calcification, as confirmed by both intraoperative gross inspection and postoperative pathological examination. In addition, preserved left ventricular structure and function were mandatory. All clinical data were collected after written informed consent was obtained.

Patients in the AS group were diagnosed in accordance with the *Expert Consensus on Clinical Pathway of Transcatheter Aortic Valve Replacement in China (2024 Edition)*. The control group included age-matched patients without AS who were admitted during the same study period. The two groups were comparable with respect to age (AS: 66 ± 3 years; control: 63 ± 3 years) and body mass index (AS: 20.32 ± 2.58; control: 21.88 ± 4.00).(Supplementary Table 1).

All patients in the AS group were younger than 70 years, presented with severe AS, and met one or more consensus-defined criteria, including the presence of surgical contraindications or high-risk factors such as prior thoracic radiotherapy, hepatic failure, diffuse severe aortic calcification, or extreme frailty. Exclusion criteria for both groups included other significant cardiovascular diseases (e.g., severe mitral valve disease or coronary artery disease requiring revascularization), uncontrolled hypertension, active infection, malignancy, or inability to provide informed consent (Table 1). The study was conducted in accordance with the principles of the Declaration of Helsinki.

**Table 1:** The characteristics of study population

Terms	AS	control	*P*
N	8	8	
age (years)	66 ± 3	63 ± 3	0.066
Sex			1.000
male	5(62.5%)	5(62.5%)	
female	3(37.5%)	3(37.5%)	
Smoking	1(12.5%)	5(62.5%)	0.121
Drinking	1(12.5%)	5(62.5%)	0.121
BMI	20.32 ± 2.58	21.88 ± 4.00	0.371

### RNA extraction, cDNA library construction, and Illumina sequencing

Total RNA was extracted from 16 samples using TRIzol reagent (Invitrogen, USA). All samples met predefined quality control criteria. Ribosomal RNA was removed from each sample using the Ribo-Zero rRNA Removal Kit (Epicenter, USA). cDNA libraries were constructed using the NEBNext^®^ Ultra™ Directional RNA Library Prep Kit for Illumina^®^ (NEB, USA) according to the manufacturer’s instructions.

The libraries were sequenced on the Illumina HiSeq 2500 platform, yielding raw sequencing reads. Sequencing quality was assessed using FastQC software by evaluating the distribution of sequencing error rates. Gene expression abundance was quantified as fragments per kilobase of exon per million mapped reads, accounting for both gene length and sequencing depth.

Ribosomal RNA was first removed from the total RNA, followed by fragmentation under controlled conditions. Double-stranded cDNA was synthesized, with strand specificity introduced during second-strand synthesis. Sequencing adapters were ligated to the cDNA fragments, and size selection was performed to obtain appropriately sized library fragments. Strand-specific polymerase chain reaction (PCR) amplification was then carried out, and the amplified libraries were subsequently purified. Library quality was assessed using standard quality control tools prior to sequencing. The final libraries were sequenced on the Illumina platform using a defined sequencing strategy.

A total of 984,613,946 raw sequencing reads were generated. Following quality filtering using fastp software, 857,601,601 clean reads were obtained, with the proportion of clean reads exceeding 74.3% across all samples. Sequencing quality was high, with Q20 scores above 96% and Q30 scores exceeding 91.1% for all libraries (Supplementary Table 2), confirming the reliability of the dataset.

### Analysis of differentially expressed transcripts and functional enrichment

Differential expression analyses of mRNAs and lncRNAs were performed using the DESeq2 package in R, and the expression profiles of circRNAs were also assessed [9]. Transcripts with an absolute log₂ fold change ≥ 1 and a *p* value ≤ 0.05 were considered significantly differentially expressed.

Functional characterization of these transcripts was carried out using Gene Ontology (GO) and Kyoto Encyclopedia of Genes and Genomes (KEGG) pathway enrichment analyses with the clusterProfiler package [10]. GO analysis was used to evaluate cellular components, biological processes, and molecular functions, whereas KEGG pathway analysis identified signaling and metabolic pathways associated with the differentially expressed genes (DEGs). A *p* value threshold of < 0.05 was applied to define statistically significant functional and pathway enrichment.

### Experimental validation

All animal experiments were conducted in accordance with the *Guide for the Care and Use of Laboratory Animals* and were approved by the Institutional Animal Care and Use Committee of Xinjiang Medical University. Sprague–Dawley (SD) rats were obtained from the Animal Experimentation Center of Xinjiang Medical University (production license number: SCXK [Xin] 2023-0001). Animals were housed in a specific pathogen-free facility under controlled environmental conditions, including a temperature of 24–26 °C, relative humidity of 40–60%, and a 14-hour light/10-hour dark cycle. Each cage accommodated five SD rats, with bedding replaced two to three times per week. The barrier environment provided 15 air changes per hour, with an airflow velocity of 0.1–0.2 m/s. Rats had free access to food and water and were acclimated for one week under a 12-hour light/12-hour dark cycle, during which general health status and behavior were routinely monitored.

The AS model was established using transverse aortic constriction (TAC). Male SD rats (40 weeks old, 500–700 g) were anesthetized with inhalational isoflurane, underwent endotracheal intubation, and were subjected to a left anterolateral thoracotomy to expose the ascending aorta. A 22G needle was positioned adjacent to the aorta to standardize the degree of constriction, and a 2 − 0 silk suture was tied to reduce the aortic lumen by approximately 70%, maintaining an estimated 30% patency. After removal of the needle, the thoracic cavity was closed in layers. Buprenorphine was administered for postoperative analgesia, and ceftazidime was used for infection prophylaxis. Postoperative monitoring included assessment of consciousness, activity levels, feeding behavior, and body weight.

At four weeks after TAC, aortic valve tissues were harvested for analysis of Nicotinamide Adenine Dinucleotide Kinase 2 (NADK2) expression. Total RNA was extracted using TRIzol reagent, and complementary DNA (cDNA) was synthesized for quantitative real-time polymerase chain reaction (qRT-PCR) using SYBR Green chemistry on a real-time PCR system. Human sequences were as follows: forward 5’-GATTACAGGCAGGCACCACTATG-3’ and reverse 5’-AGGCAGATCACAAGGTCAGGAG-3’; rat primer sequences were forward 5’-CTTCAGTATCCGAGAGCCAATAGC-3’ and reverse 5’-CCCAACACCGAGAACGAACAC-3’. For protein analysis, tissue lysates were subjected to Western blotting using anti-NADK2 (F5 × 1 V) and anti-GAPDH (A19056) antibodies. Protein band intensities were quantified using ImageJ software.

### Weighted gene correlation network analysis

Weighted Gene Correlation Network Analysis (WGCNA) was performed using the top 8,000 differentially expressed genes ranked according to *p*-values [11]. Gene co-expression modules were constructed after determining the optimal soft-thresholding power using the pickSoftThreshold function. Candidate power values ranging from 1 to 30 were evaluated based on average connectivity and module independence, with a power considered acceptable when module independence exceeded 0.9. Modules were required to contain a minimum of 30 genes, and distinct module colors were automatically assigned for visualization and differentiation using the WGCNA package in R.

### Profiles of immune and stromal cell infiltration

To address the heterogeneity inherent to bulk tissue sequencing data, the composition of immune and stromal cells was computationally estimated using the xCell algorithm, which integrates gene expression signatures to infer the relative enrichment of 64 distinct cell types [12]. Normalized expression matrices from the present dataset were analyzed using the xCell platform (https://xcell.ucsf.edu/). Enrichment scores were calculated for immune and stromal cell populations, and comparisons between patients with AS and control groups were performed using the Wilcoxon test to identify differentially enriched cell subsets.

### Construction of Protein-protein Interaction (PPI) network and gene-immune cell correlation analysis

To construct a high-confidence PPI network and enhance topological interpretability, a more stringent statistical threshold was applied to the DEG dataset. Only DEGs with a false discovery rate (FDR) < 0.05 were selected for analysis using the STRING database (https://string-db.org/). The minimum interaction confidence score was set to > 0.40, and the resulting interaction network was visualized to identify hub genes and functional clusters [13].

Hub genes identified from the PPI network were subsequently examined for associations with immune cell infiltration. Spearman’s correlation coefficients were calculated between hub gene expression levels and the estimated abundances of the top 15 immune cell types. Correlation results were visualized using a heatmap generated with the Sangerbox platform (http://www.sangerbox.com/) [14].

## Results

### Differential expression analysis of mRNAs and functional enrichment analysis

**Fig. 1:**
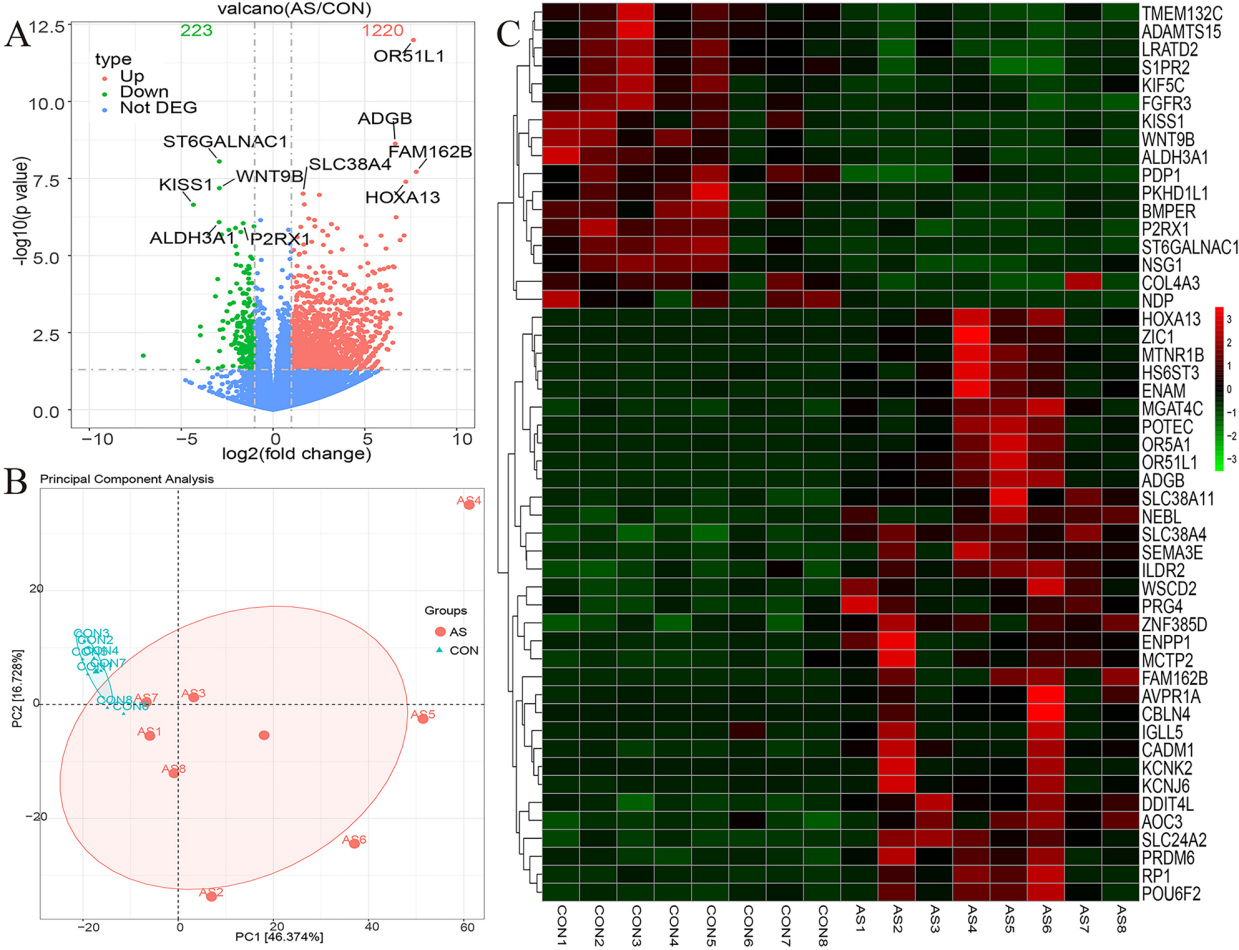
Volcano plot, principal component analysis, and heatmap of differentially expressed mRNAs in the AS and control group. **A** Volcano plot. **B** Principal component analysis. **C** Heatmap.

Differential expression analysis identified 1,443 genes that met the predefined thresholds of |log2 (fold change)| ≥ 1 and *p* ≤ 0.05, of which 1,220 were upregulated and 223 downregulated in AS compared with controls (Fig. 1A). Principal component analysis demonstrated clear separation between the AS and control groups, indicating distinct global transcriptomic profiles (Fig. 1B). A heatmap of the top 50 DEGs highlighted prominent transcripts, including *TMEM132C*,* ADAMTS15*,* LRATD2*,* S1PR2*,* KIF5C*,* HOXA13*,* ZIC1*,* MTNR1B*,* HS6ST3*, and *ENAM* (Fig. 1C). 

**Fig. 2:**
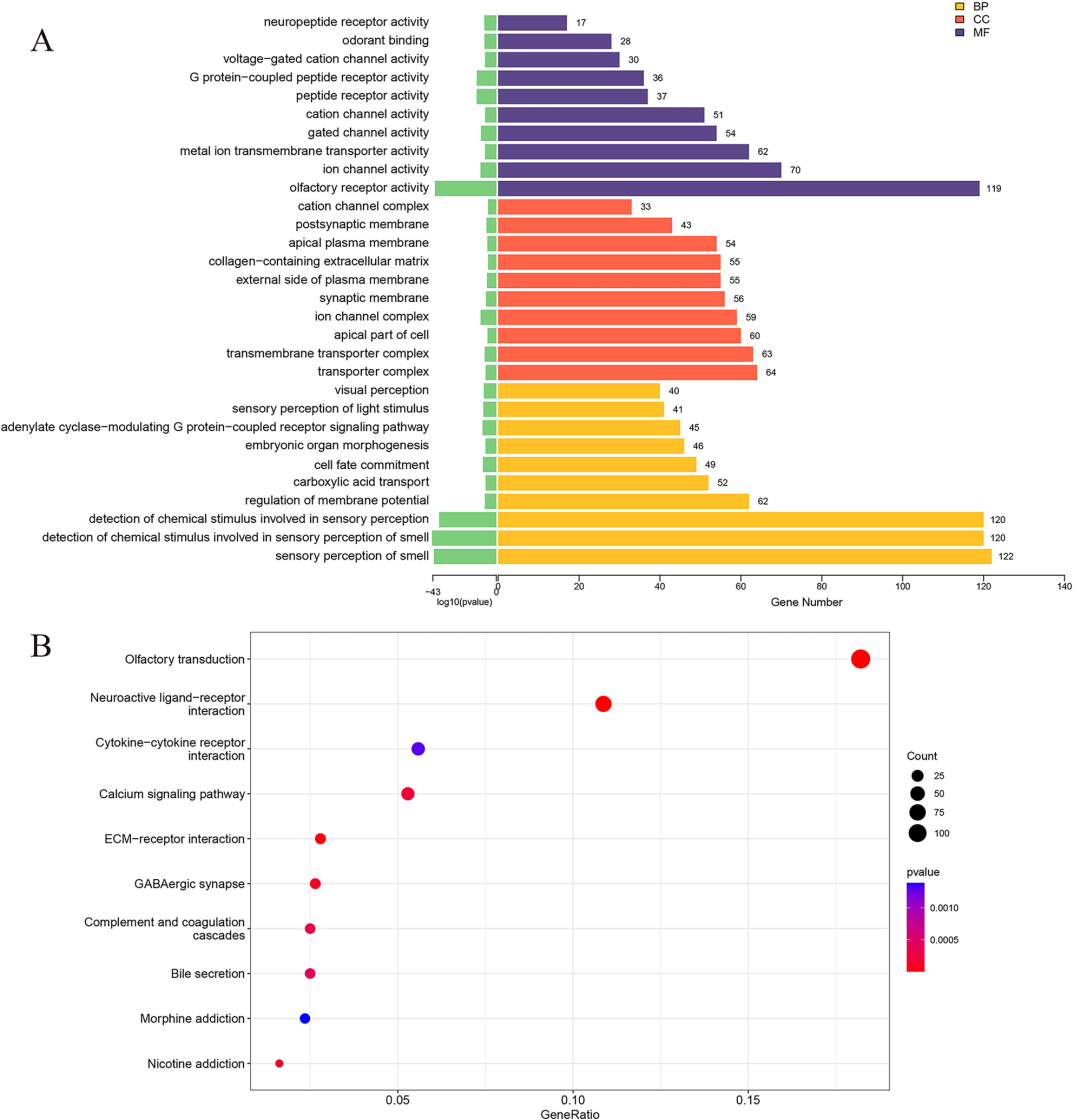
Functional enrichment analysis of the differentially expressed mRNAs. **A** GO enrichment analysis. **B** KEGG pathway enrichment analysis.

Functional enrichment analysis revealed that these DEGs were significantly associated with biological processes such as sensory perception of smell, regulation of membrane potential, and carboxylic acid transport. Notably, enrichment of olfactory transduction–related pathways was observed, a finding of interest given emerging evidence that ectopic olfactory receptors may contribute to vascular inflammation and macrophage activation (Fig. 2A). In addition, enrichment of calcium signaling pathways and ion channel–related functions was consistent with the pathological calcification processes characteristic of AS, in which calcium dysregulation promotes osteogenic differentiation of valvular interstitial cells (Fig. 2B).

**Fig. 3:**
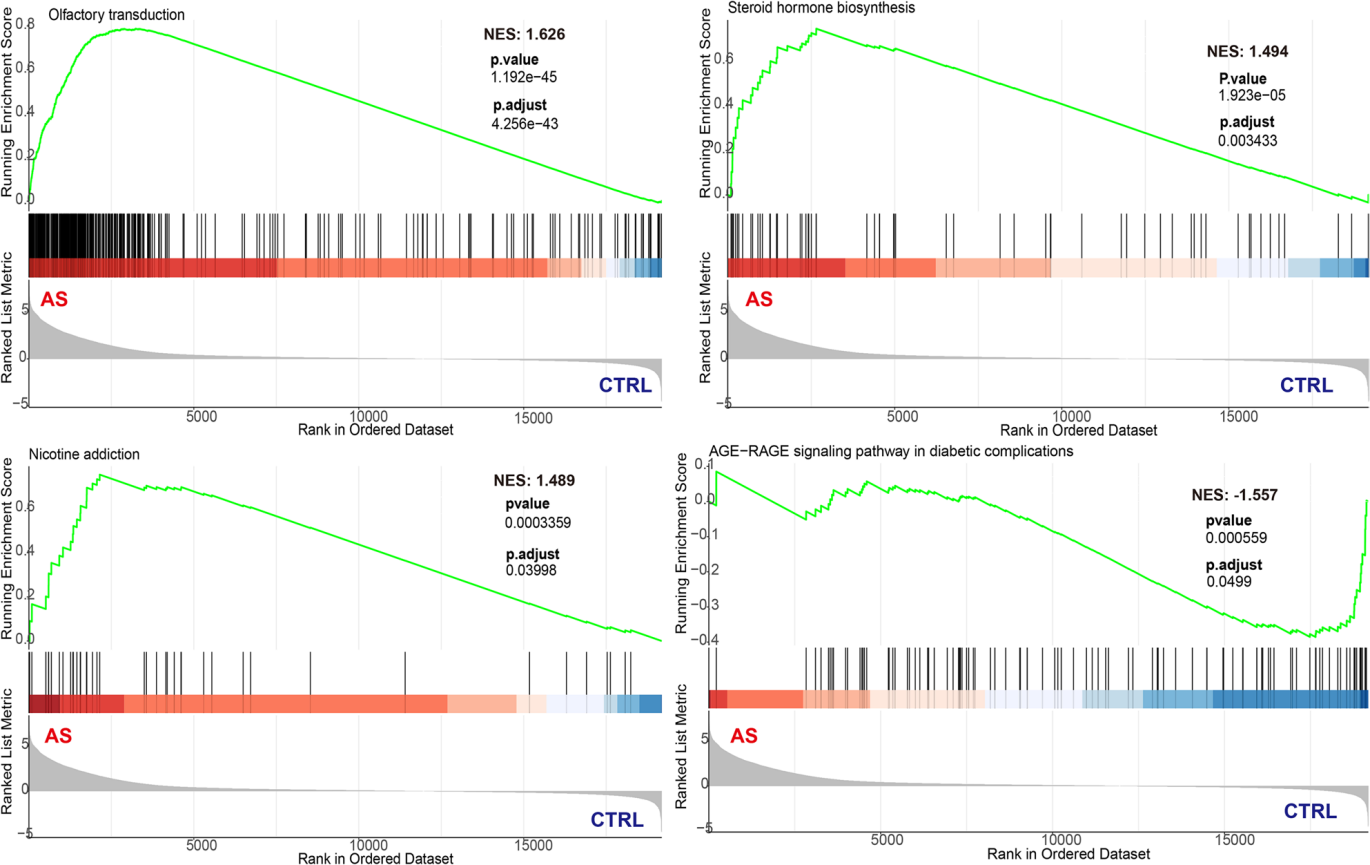
GSEA of significantly enriched pathways

Gene set enrichment analysis further identified four significantly enriched pathways: olfactory transduction, steroid hormone biosynthesis, nicotine addiction, and advanced glycation end product–receptor for advanced glycation end product signaling in diabetic complications (Fig. 3).

### Differential expression analysis of lncRNAs and functional enrichment analysis

**Fig. 4:**
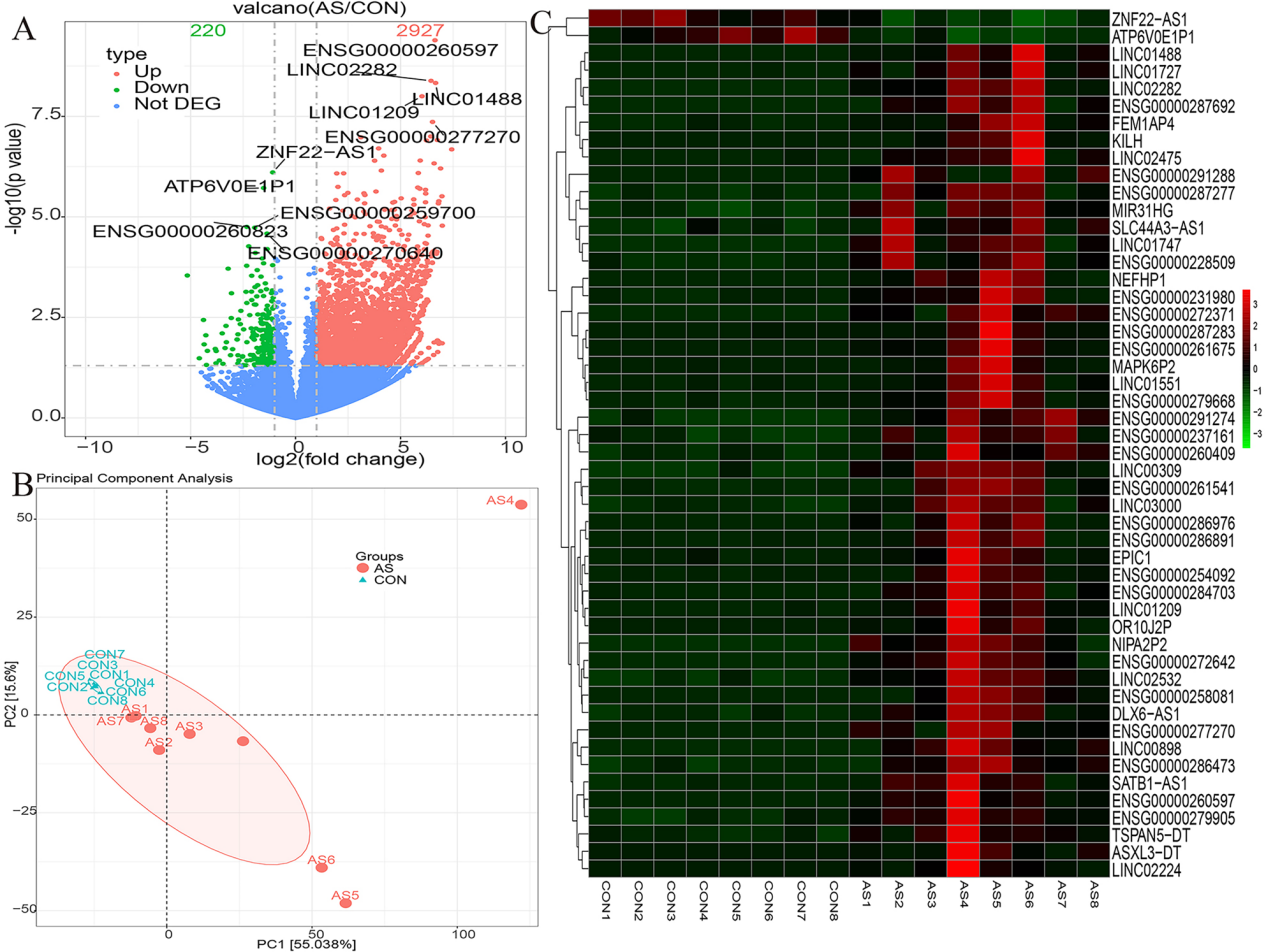
Volcano plot, principal component analysis, and heatmap of significantly differentially expressed lncRNAs between the AS group and the control group. **A** Volcano plot. **B** Principal component analysis. **C** Heatmap.

Analysis of lncRNAs identified 3,147 differentially expressed lncRNAs based on the same statistical thresholds, including 2,927 upregulated and 220 downregulated transcripts (Fig. 4A). PCA demonstrated clear separation between AS and control groups, indicating distinct lncRNA expression profiles (Fig. 4B). A heatmap of the top 50 differentially expressed lncRNAs highlighted representative transcripts, including *ZNF22-AS1*,* ATP6V0E1P1*,* ENSG00000287692*,* LINC01727*,* LINC02282*,* LINC01488*, and *FEM1AP4* (Fig. 4C).

**Fig. 5:**
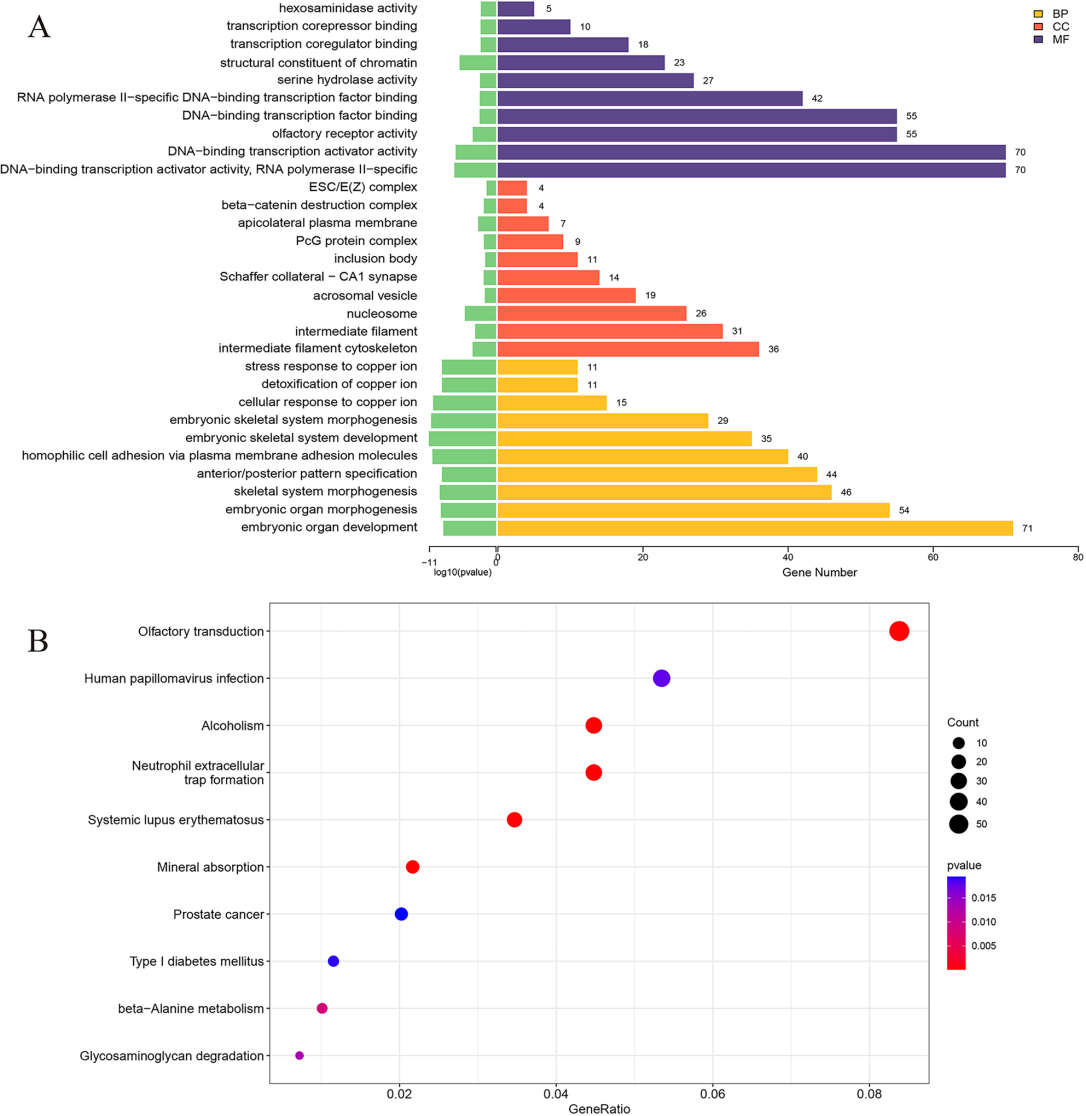
Functional enrichment analysis of target genes associated with differentially expressed lncRNAs. **A** GO enrichment analysis. **B** KEGG pathway enrichment analysis.

Target gene enrichment analysis revealed significant associations with biological processes related to embryonic organ development, skeletal system morphogenesis, and anterior–posterior pattern specification. Enrichment was also observed for cellular components such as the intermediate filament cytoskeleton, nucleosome, and acrosomal vesicle, as well as for molecular functions including olfactory receptor activity, DNA-binding transcription activator activity, and serine hydrolase activity (Fig. 5A). Pathway analysis further indicated enrichment in olfactory transduction, neutrophil extracellular trap formation, mineral absorption, and beta-alanine metabolism (Fig. 5B).

### Differential expression analysis of circRNAs and functional enrichment analysis

**Fig. 6:**
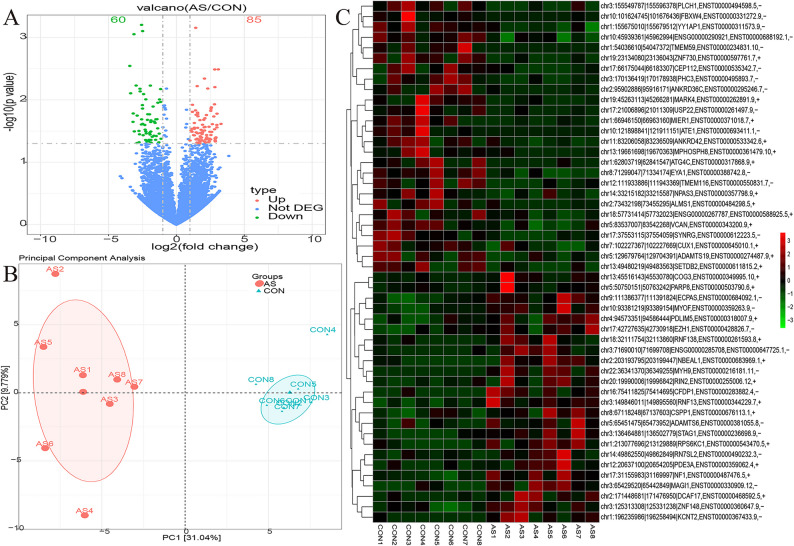
Volcano plot, principal component analysis, and heatmap of significantly differentially expressed circRNAs between the AS group and the control group. **A** Volcano plot. **B** Principal component analysis. **C** Heatmap.

Using the predefined thresholds of |log2 (fold change)| ≥ 1 and *p*-value ≤ 0.05, a total of 145 differentially expressed circRNAs were identified, comprising 85 upregulated and 60 downregulated transcripts (Fig. 6A). PCA based on circRNA expression profiles demonstrated clear separation between AS and control groups (Fig. 6B). In addition, a heatmap illustrating the top 50 most differentially expressed circRNAs was generated (Fig. 6C).

**Fig. 7:**
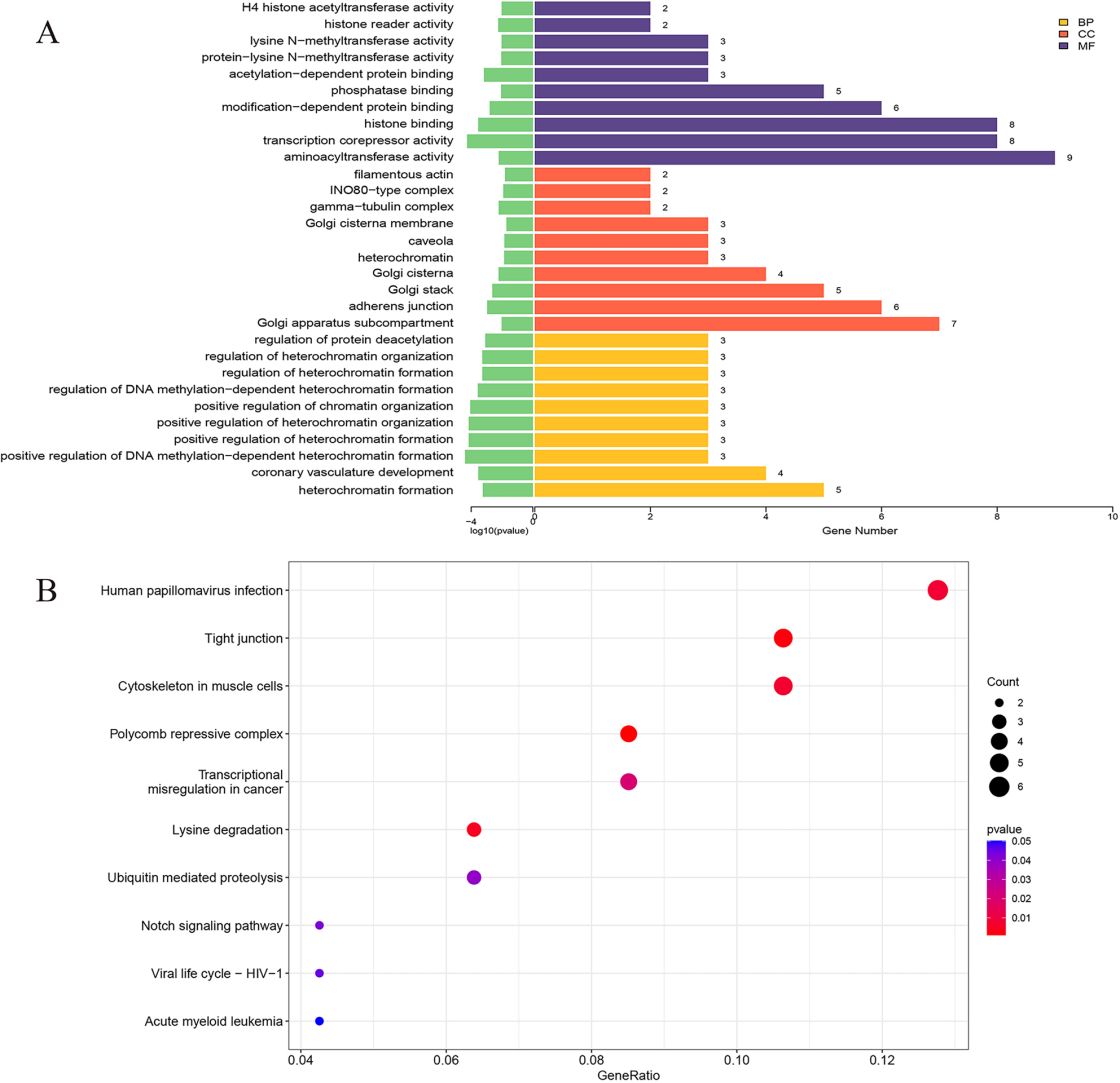
Functional enrichment analysis of source genes of differentially expressed circRNAs. **A** GO enrichment analysis. **B** KEGG pathway enrichment analysis.

Functional enrichment analysis of the source genes corresponding to these differentially expressed circRNAs revealed significant associations with multiple biological processes, including heterochromatin formation, coronary vasculature development, and regulation of protein deacetylation. Enrichment was also observed for cellular components such as the Golgi apparatus subcompartment, adherens junctions, and heterochromatin. Furthermore, these source genes were enriched in molecular functions including aminoacyl transferase activity, transcription corepressor activity, and histone binding (Fig. 7A). Pathway enrichment analysis indicated involvement in tight junction regulation, cytoskeleton organization in muscle cells, lysine degradation, ubiquitin-mediated proteolysis, and the Notch signaling pathway (Fig. 7B).

### Immune infiltration, PPIs, and gene-immune cell correlations in AS

**Fig. 8:**
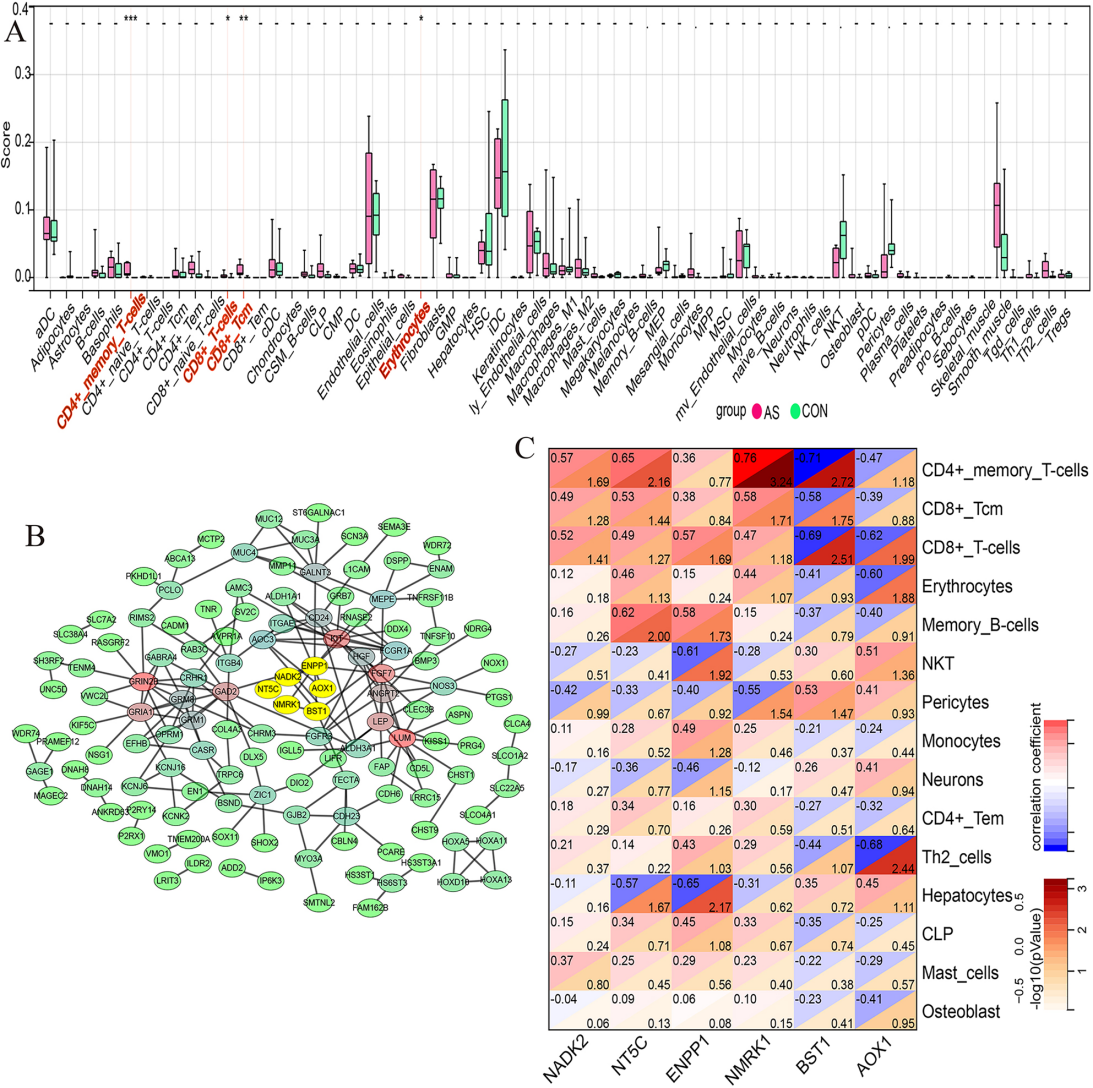
Analysis of immune infiltration, protein–protein interactions, and gene–immune cell correlations in aortic stenosis. **A** Immune infiltration analysis of aortic stenosis samples. The bar plot presents the relative abundance of immune cell types. **B** Protein–protein Interaction (PPI) network constructed from differentially expressed genes with a False Discovery Rate (FDR) < 0.05. Six genes enriched in the nicotinate and nicotinamide metabolism pathway are highlighted. **C** Correlation heatmap showing associations between the six genes enriched in the nicotinate and nicotinamide metabolism pathway and the top 15 immune cell types ranked by *p*-value. The heatmap illustrates the correlation coefficients, with red indicating positive correlations and blue indicating negative correlations. The analysis highlights the correlations between these genes and infiltrating immune cells, with a focus on *NADK2*.

To characterize the immunological landscape of AS, the cellular composition of valve tissues was deconvoluted using the xCell algorithm. As shown in Fig. 8A, the immune microenvironment of AS valves differed markedly from that of control tissues and was characterized by increased infiltration of adaptive immune cells. In particular, significantly higher proportions of CD4⁺ memory T cells and CD8⁺ T cells, including central memory CD8⁺ T cell subsets (CD8⁺ Tcm), were observed. The enrichment of memory T cell phenotypes is consistent with a chronic, antigen-driven adaptive immune response, potentially initiated by neoantigens released during valvular endothelial injury or lipid oxidation, thereby sustaining inflammation and promoting valvular remodeling.

To investigate molecular features associated with this immune profile, a PPI network was constructed using the DEGs (Fig. 8B). This analysis identified a central cluster of six genes involved in nicotinate and nicotinamide metabolism, including *NADK2*, *NT5C*, *ENPP1*, *NMRK1*, *BST1*, and *AOX1*. Given the established role of NAD⁺ metabolism in immune cell energy homeostasis and survival, correlations between the expression of these metabolic genes and immune cell infiltration were further examined (Fig. 8C). Notably, *NADK2* expression showed strong positive correlations with the abundance of CD4⁺ memory T cells (*r* = 0.57), CD8⁺ Tcm (*r* = 0.49), and CD8⁺ T cells (*r* = 0.52). These associations suggest that upregulation of *NADK2* may support metabolic adaptation of T cells within the hypoxic and inflammatory environment of the calcified aortic valve, thereby contributing to the persistence of adaptive immune responses in AS.

### Elevated NADK2 expression in aortic stenosis: validation in human and rat models

**Fig. 9:**
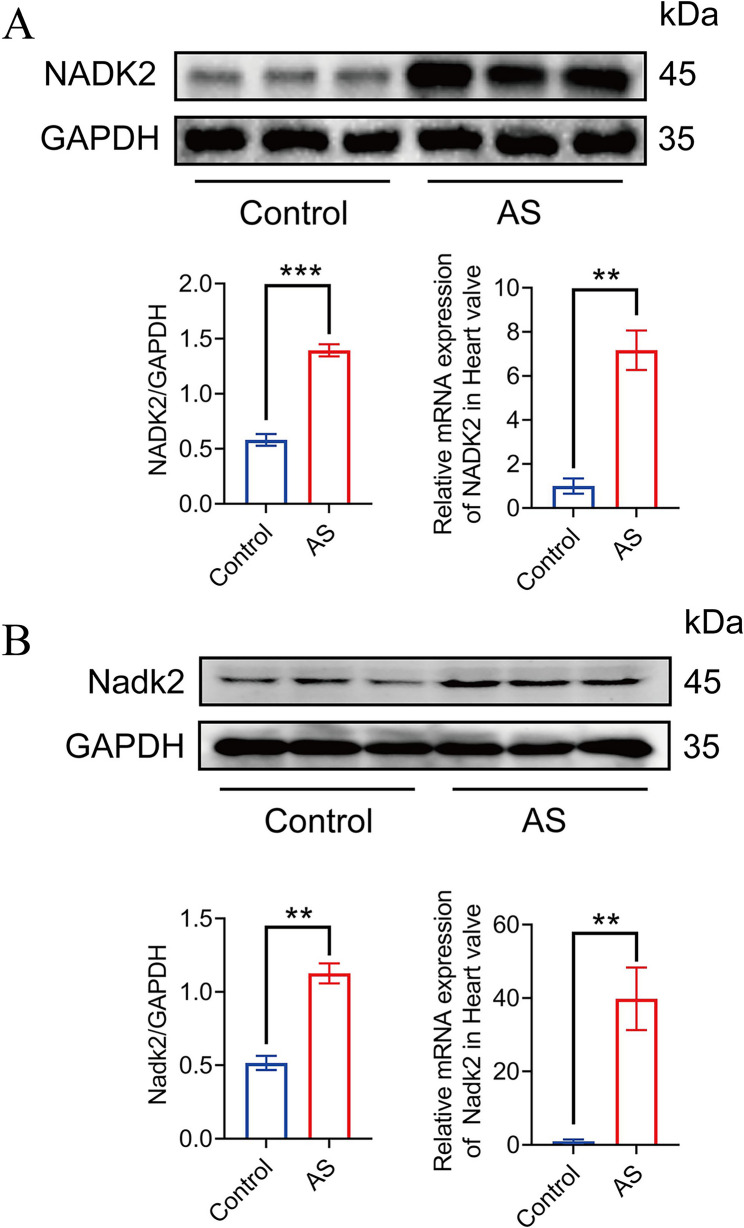
Elevated NADK2 expression in Aortic Stenosis (AS) in human and rat models. **A** Western blotting and qPCR analysis of NADK2 expression in human samples. The left panel shows representative Western blot images of NADK2 and GAPDH (loading control) in control and AS samples. The right panel presents quantification of NADK2 protein expression normalized to GAPDH (NADK2/GAPDH ratio) and relative NADK2 mRNA expression in aortic valve tissues. **B** Western blotting and qPCR analysis of NADK2 expression in the rat AS model. The left panel shows representative Western blot images of NADK2 and GAPDH (loading control) in control and AS rats. The right panel presents quantification of NADK2 protein expression normalized to GAPDH (NADK2/GAPDH ratio) and relative NADK2 mRNA expression in aortic valve tissues. Data are presented as mean ± SEM. ** *p* < 0.01 vs. control; *** *p* < 0.001 vs. control

NADK2 expression in AS was evaluated using western blotting and quantitative polymerase chain reaction (qPCR) analyses in both human and rat AS samples. In human valve tissues (Fig. 9A), western blot analysis demonstrated a significant increase in NADK2 protein expression in patients with AS compared with controls, as confirmed by densitometric quantification normalized to GAPDH. Consistent with these findings, qPCR analysis revealed significant upregulation of *NADK2* mRNA in aortic valve tissues from patients with AS.

Comparable results were observed in the rat AS model (Fig. 9B). NADK2 protein expression was significantly elevated in AS rats relative to control animals, and qPCR analysis confirmed marked upregulation of *Nadk2* mRNA in rat aortic valve tissues (Supplementary Figs. 1, 2, 3, and 4).

### WGCNA analysis of gene expression in patients with AS

**Fig. 10:**
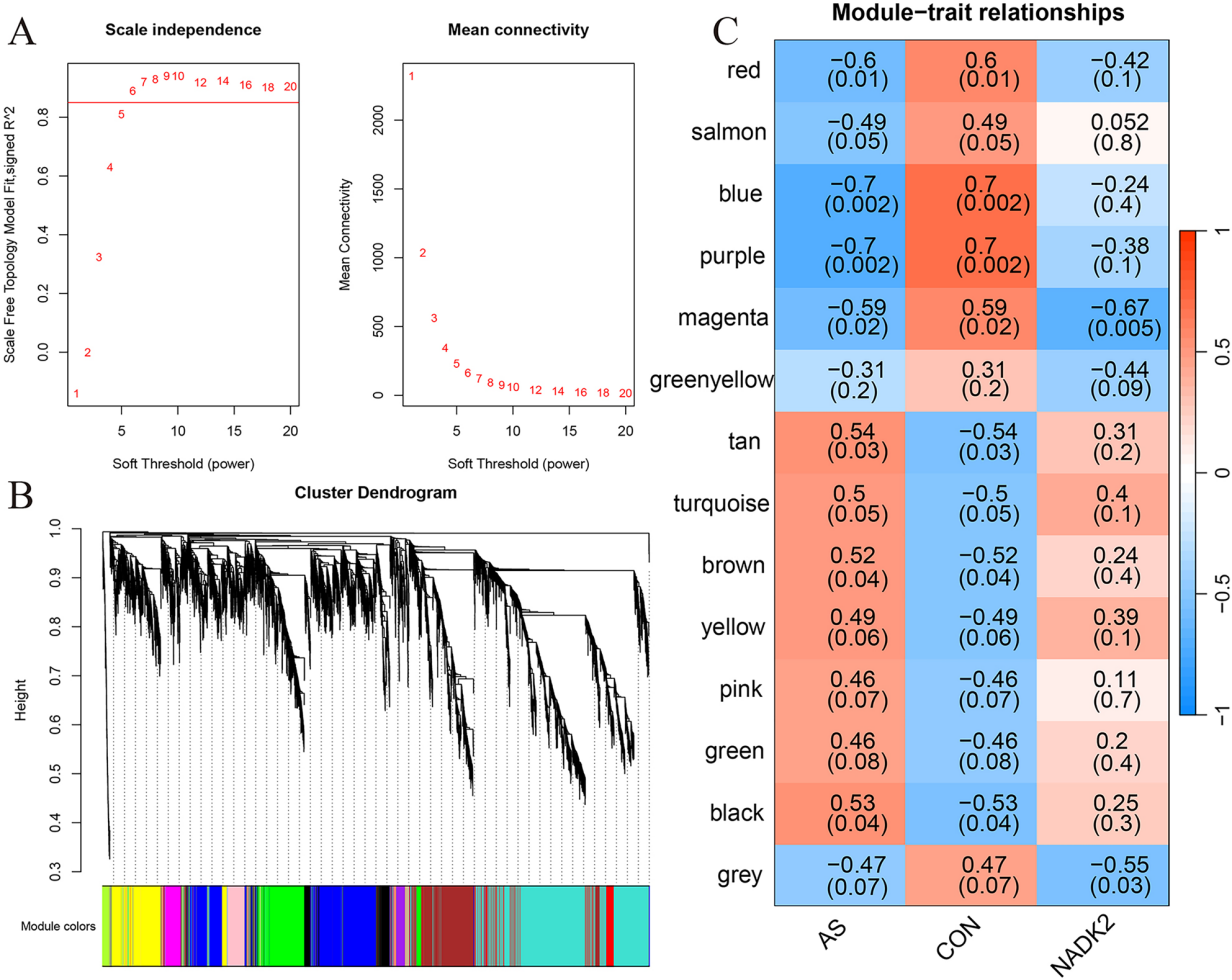
Weighted Gene Correlation Network Analysis (WGCNA) of gene expression in patients with aortic stenosis. **A** Determination of the optimal soft-thresholding power for network construction. Scale independence and mean connectivity were evaluated. **B** Hierarchical clustering dendrogram of gene modules, with each color representing a distinct module. **C** Heatmap of module–trait relationships showing correlation coefficients and corresponding *p*-values between gene modules and traits (AS, CON, and NADK2)

WGCNA was performed on transcriptomic data from patients with AS, with a specific focus on *NADK2*. During network construction (Fig. 10A), the optimal soft-thresholding power was determined to be 6, at which point the scale-free topology fit index reached 0.9 and mean connectivity stabilized, indicating an appropriate network structure. Hierarchical clustering identified distinct gene modules, each assigned a unique color to represent specific co-expression patterns (Fig. 10B).

Module–trait correlation analysis showed that the magenta module exhibited the strongest positive association with *NADK2* expression (*r* = 0.59, *p* = 0.02; Fig. 10C, Supplementary Table 3). These findings suggest that the magenta module may have a relevant role in AS pathogenesis, particularly in relation to *NADK2*.

### Functional enrichment analysis of *NADK2*-related AS genes.

**Fig. 11:**
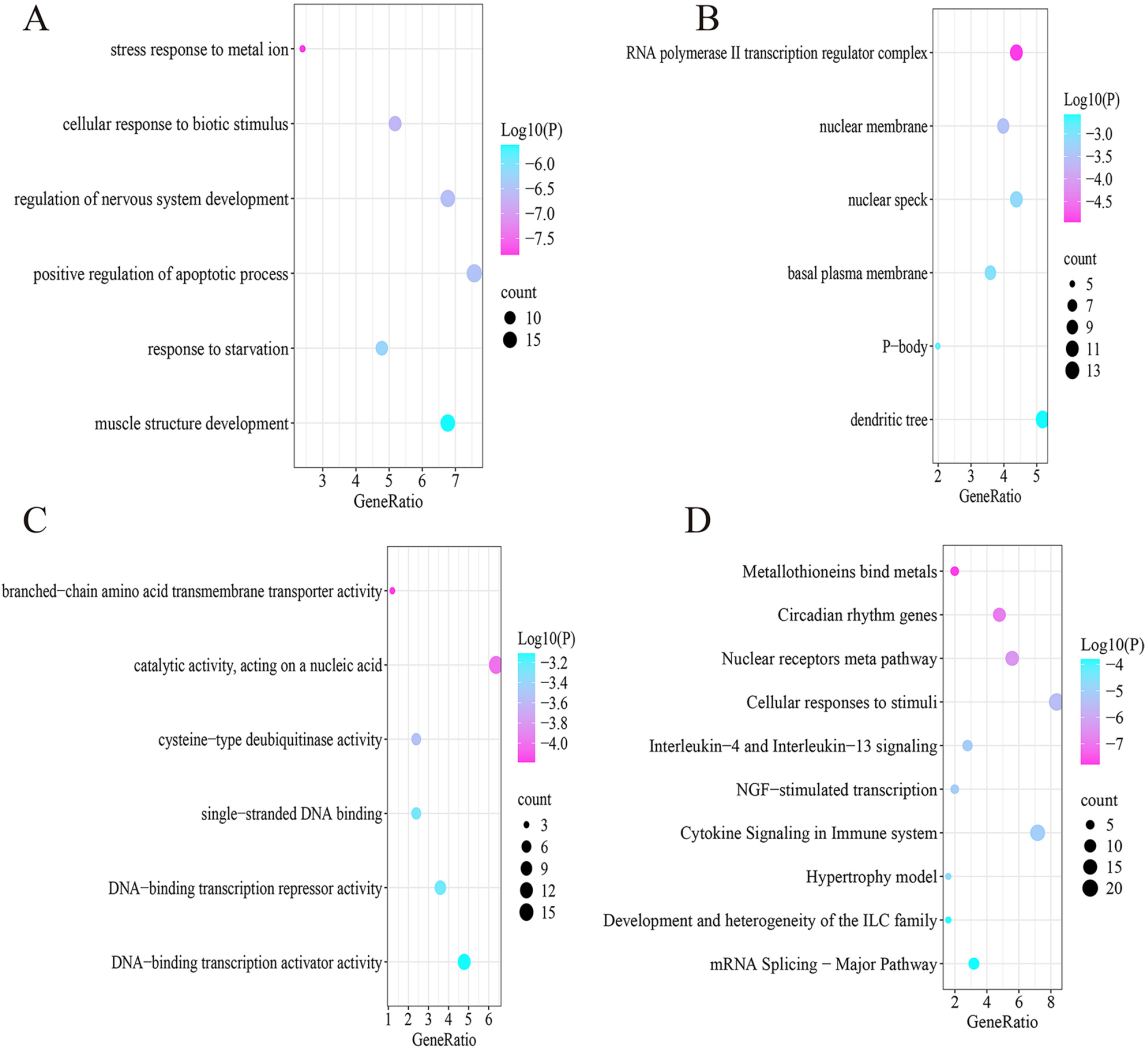
Functional enrichment analysis of NADK2-related genes in aortic stenosis. **A** Biological process enrichment analysis. **B** Cellular component enrichment analysis. **C** Molecular function enrichment analysis. **D** Pathway enrichment analysis.

Functional enrichment analysis of genes within the magenta module, which showed strong correlations with *NADK2* expression, revealed significant enrichment in biological processes including stress responses to metal ions, cellular responses to biotic stimuli, and regulation of nervous system development (Fig. 11A). These genes were also significantly enriched in cellular components such as the RNA polymerase II transcription regulator complex, the nuclear membrane, and nuclear speckles (Fig. 11B).

Analysis of molecular functions indicated enrichment in branched-chain amino acid transmembrane transporter activity, catalytic activity acting on nucleic acids, and cysteine-type deubiquitinase activity (Fig. 11C). Pathway enrichment analysis further demonstrated significant involvement in metallothionein-mediated metal binding, circadian rhythm regulation, nuclear receptor signaling, cellular responses to environmental stimuli, and interleukin-4 and interleukin-13 signaling pathways (Fig. 11D).

## Discussion

AS is a prevalent cardiac disorder characterized by progressive narrowing of the aortic valve, leading to obstruction of blood flow from the left ventricle into the aorta and systemic circulation [15]. This hemodynamic burden contributes to clinical manifestations such as exertional dyspnea, angina, syncope, and eventual progression to heart failure [16]. AS is associated with substantial morbidity and mortality, particularly among older adults, and remains a major focus of contemporary cardiology and cardiovascular research [17]. Etiologically, AS arises either from congenital valve abnormalities, such as bicuspid aortic valve, or from age-related degenerative calcification of a trileaflet valve [18]. Its pathophysiology reflects a complex interplay among genetic susceptibility, environmental influences, and hemodynamic stress, highlighting the need for continued investigation into the molecular mechanisms driving disease progression and for the identification of novel therapeutic targets [19].

In this study, a comprehensive transcriptomic analysis encompassing mRNAs, lncRNAs, and circRNAs was undertaken to clarify their potential contributions to AS. High-throughput RNA sequencing was applied to characterize differential RNA expression profiles in patients with AS, addressing existing gaps in understanding RNA-mediated regulatory interactions in this disease. Substantial alterations in the expression of numerous RNA species were identified, providing insight into molecular drivers of AS and highlighting potential biomarkers for early diagnosis and therapeutic intervention. Integration of transcriptomic data with immune cell infiltration patterns and metabolic pathway analyses further offered a broader perspective on AS pathogenesis, underscoring the relevance of coordinated immune–metabolic regulation.

The transcriptomic landscape of AS was marked by distinct expression profiles, including 1,443 differentially expressed mRNAs and 3,147 lncRNAs, with enrichment in pathways related to sensory perception, ion transport, and developmental processes. Notably, olfactory transduction emerged as a top enriched pathway among both DEGs and lncRNA target genes. This finding is consistent with growing evidence that olfactory receptors exert non-olfactory functions in vascular tissues and contribute to inflammatory signaling [20]. For example, receptors such as OLFR2/OR6A2 expressed in macrophages have been shown to activate NLRP3 inflammasomes in response to lipid peroxidation products, leading to interleukin-1β–driven inflammation [21]. In light of the upregulation of NLRP3-related genes observed in the present dataset, this mechanism may be relevant to AS pathobiology.

Enrichment of calcium signaling pathways further supports the central role of calcium dysregulation in valvular calcification. The observed upregulation of osteogenic regulators such as *RUNX2* and *CACNA1C* is consistent with established mechanisms whereby calcium signaling promotes osteogenic differentiation of valvular interstitial cells in calcified valves [22]. Developmental pathways were also prominent in the lncRNA-associated analyses, with target genes enriched in embryonic organogenesis and skeletal morphogenesis. These findings suggest that reactivation of fetal or developmental programs may contribute to pathological remodeling of the aortic valve. Prior studies have demonstrated that lncRNAs such as MEG3 can modulate Wnt/β-catenin signaling and promote osteoblastic differentiation of valvular interstitial cells [23]. Although integration of mRNA–lncRNA networks provided novel insights, functional validation of candidate transcripts, including *TMEM132C* and *ZNF22-AS1*, remains necessary to confirm their specific roles in these pathways. Collectively, these observations support the concept of AS as a disorder driven by maladaptive signaling across sensory, metabolic, and developmental axes.

Immune cell infiltration also emerged as a prominent feature of AS in this analysis, with significant associations observed between DEGs and specific immune cell subsets. The immune microenvironment of AS valves was characterized by prominent infiltration of adaptive immune cells, particularly CD4⁺ memory T cells and CD8⁺ T cells. These findings align with prior reports indicating that clonal expansion of T cells and immune-mediated calcification are hallmarks of AS progression [24]. The oligoclonal nature of infiltrating T cells suggests antigen-specific immune activation, potentially directed against valvular antigens modified by chronic inflammation or oxidative stress [25].

PPI network analysis identified a cluster of six genes enriched in the nicotinate and nicotinamide metabolism pathway, which plays a central role in nicotinamide adenine dinucleotide (NAD⁺) biosynthesis and regulates mitochondrial respiration, redox balance, and immune function [26]. Among these genes, *NADK2*, encoding a mitochondrial NAD kinase, showed the strongest correlation with immune cell infiltration [27]. Although *NADK2* has not previously been linked to AS, it is known to maintain mitochondrial NADPH pools required for antioxidant defense and has been implicated in T cell function, suggesting a potential role in coordinating oxidative stress responses and immune crosstalk within valvular tissue [28]. Based on integrated transcriptomic, network, and immune correlation analyses, *NADK2* emerges as a candidate regulator of immune–metabolic interactions in AS. Cross-species validation further demonstrated consistent upregulation of *NADK2* at both the transcript and protein levels in human valve tissues and in the TAC rat model. Nonetheless, these findings remain associative, and targeted functional perturbation studies, such as gene knockdown or overexpression, will be required to establish mechanistic causality.

Weighted gene correlation network analysis additionally identified a magenta module enriched for genes involved in inflammatory signaling and extracellular matrix remodeling. This observation is consistent with evidence that impaired *NADK2* function disrupts mitochondrial metabolism [29]. Co-expression of *NADK2* with metalloproteinases and chemokines within this module suggests a coordinated regulatory network linking mitochondrial NADPH balance to immune cell recruitment and fibrotic remodeling. These results align with emerging paradigms in cardiovascular disease research, in which immune–metabolic cross-talk is increasingly recognized as a central pathogenic mechanism.

Several limitations of this study warrant consideration. First, the relatively small size of the human cohort (*n* = 8 per group) limited the ability to perform multivariable analyses adjusting for potential clinical confounders, such as medication use or detailed metabolic profiles. This limitation was partially mitigated through strict exclusion criteria and validation of key findings in an independent rat model. Second, transcriptomic analyses were performed on bulk valve tissues. Although immune infiltration was estimated using the xCell algorithm, such approaches reflect averaged tissue signals and lack the cellular resolution achievable with single-cell RNA sequencing. We did not perform functional perturbation experiments (e.g., gain- or loss-of-function studies) to definitively prove causality. Finally, while robust associations between *NADK2*, immune infiltration, and AS pathology were demonstrated across species, the present study remains correlative in nature. Functional studies using gene-edited animal models and primary valvular interstitial cell systems will be essential to fully elucidate the mechanistic role of *NADK2* in valvular calcification and immune regulation.

## Conclusion

In summary, this study identified substantial alterations in mRNA, lncRNA, and circRNA expression profiles in AS, thereby enhancing current understanding of the molecular mechanisms underlying this disease. The findings underscore the involvement of immune regulation and RNA-mediated signaling pathways in AS pathogenesis and highlight potential molecular targets for therapeutic exploration. Future studies should prioritize experimental validation of these observations and further assessment of their clinical relevance, with the overarching aim of improving diagnostic strategies and therapeutic interventions for the management of AS.

## Supplementary Information

Supplementary Material 1.

Supplementary Material 2.

Supplementary Material 3.

Supplementary Material 4.

Supplementary Material 5: Supplementary Figure 1. Raw western blot data for GAPDH (human samples).

Supplementary Material 6: Supplementary Figure 2. Raw western blot data for GAPDH (rat samples).

Supplementary Material 7: Supplementary Figure 3. Raw western blot data for NADK2 (rat samples).

Supplementary Material 8: Supplementary Figure 4. Raw western blot data for NADK2 (human samples).

## Data Availability

All data generated or analyzed during this study are included in this article. Further enquiries can be directed to the corresponding author.
