# Supplementary material for: Whole-transcriptome analysis of Aortic Stenosis reveals dysregulated RNA networks, immune cell infiltration, and NADK2 as a candidate regulator
Source: Hereditas. 2026 Apr 17;163:68. doi: 10.1186/s41065-026-00675-w (PMC13224427; doi:10.1186/s41065-026-00675-w)
Supplement: Supplementary file 1 — Supplementary Material 1. [file 41065_2026_675_MOESM1_ESM.pdf]

Oracle Translation(Shenzhen)Co.,Ltd.

CERTIFICATE OF COPYEDITING

TO WHOMSOEVER IT MAY CONCERN

This is to certify that the manuscript listed below has been edited for English language, grammar, punctuation, spelling, and overall style by one or more of our highly qualified native English-speaking editors.

Manuscript Title:

Whole Transcriptome Profiling of Aortic Stenosis Identifies Dysregulated RNA Networks, Immune Infiltration, and NADK2 as a Pathogenic Driver

Authors:

Yi-tong Ma, Xiang Xie

Date Issued:

Nov.19, 2025

Certificate Number:

20250920369992

Signature of Proofreading Expert:

This document certifies that the manuscript listed above was edited for English language, grammar, punctuation, spelling, and overall style by one or more of the highly qualified native English-speaking editors at Oracle Translation (Shenzhen) Co., Ltd.. Neither the research content nor the authors' intentions were altered in any way during the editing process. Documents receiving this certification are suitable for publication in terms of English language editing; however, the author has the right to accept or reject our suggestions and changes. If you have any questions or concerns about this edited document, please contact us at info@oracle-trans.com.

Oracle Translation (Shenzhen) Co., Ltd. provides a range of editing, translation, and manuscript services for researchers and publishers throughout the world. Our top-quality PhD editors are native English-speakers from the top universities of English-speaking countries. Our editors possess the highest qualifications to edit research papers written by non-native English speakers. For more information about our company, please visit www.oracle-trans.com.
