# Supplementary material for: Whole-transcriptome analysis of Aortic Stenosis reveals dysregulated RNA networks, immune cell infiltration, and NADK2 as a candidate regulator
Source: Hereditas. 2026 Apr 17;163:68. doi: 10.1186/s41065-026-00675-w (PMC13224427; doi:10.1186/s41065-026-00675-w)
Supplement: Supplementary file 2 — Supplementary Material 2. [file 41065_2026_675_MOESM2_ESM.docx]

Supplementary Table 1. Comparison of Baseline Characteristics Between the Two Groups

| **Variable** | **Control group** | **Case group** | *t/*Z/χ^2^ | *P* |
| --- | --- | --- | --- | --- |
| **Demographics** |  |  |  |  |
| Age (years, mean±SD) | 62.88±3.357 | 66.25±3.412 | 1.994 | 0.066 |
| Gender [Male, n(%)] | 5(62.5) | 5(62.5) | 0.000 | 1.000 |
| Smoking [Yes, n(%)] | 1(12.5) | 5(62.5) | 2.400 | 0.121 |
| Alcohol consumption [Yes, n(%)] | 1(12.5) | 5(62.5) | 2.400 | 0.121 |
| BMI(kg/m^2^,‾*x*±s) | 21.88±4.000 | 20.32±2.570 | -0.924 | 0.371 |
| **Comorbidities** |  |  |  |  |
| Hypertension [Yes, n(%)] | 3(37.5) | 4(50.0) | 0.000 | 1.000 |
| Diabetes mellitus [Yes, n(%)] | 0 | 0 |  |  |
| Coronary heart disease [Yes, n(%)] | 0 | 3(37.5) | 1.641 | 0.200 |
| Atrial fibrillation [Yes, n(%)] | 0 | 1(12.5) | 0.000 | 1.000 |
| **Medication use** |  |  |  |  |
| Statins [Yes, n(%)] | 2(25.0) | 3(37.5) | 0.000 | 1.000 |
| Aspirin [Yes, n(%)] | 1(12.5) | 3(37.5) | 0.333 | 0.564 |
| ACEI/ARB [Yes, n(%)] | 6(75.0) | 4(50.0) | 0.267 | 0.606 |
| Beta-blockers [Yes, n(%)] | 0 | 2(25.0) | 0.571 | 0.450 |
| **Cardiac structure and function** |  |  |  |  |
| Aortic sinus diameter [mm, M(P25, P75)] [mm,M(P_25,_ P_75_)] | 46.50(37.75, 54.25) | 35.00(30.50, 36.00) | -2.321 | **0.020** |
| Left atrial diameter (mm, mean±SD) | 42.75±4.23 | 40.63±6.28 | -0.794 | 0.441 |
| Left ventricular end-diastolic diameter (mm, mean±SD) | 65.88±11.70 | 55.5±11.35 | -1.800 | 0.093 |
| Interventricular septal thickness (mm, mean±SD) | 10.63±2.45 | 12.00±2.27 | 1.166 | 0.263 |
| Left ventricular posterior wall thickness (mm, mean±SD) | 10.5±2.20 | 11.38±2.00 | 0.832 | 0.419 |
| Effective orifice area (cm², M(P25, P75)) | 4.75(4.45, 4.88) | 0.78(0.63,0.80) | -3.373 | **0.001** |
| Peak systolic transvalvular velocity (m/s, mean±SD)(m/s,‾*x*±s) | 2.26±0.41 | 4.34±0.98 | 5.550 | **<0.001** |
| LVEF(%,‾*x*±s) | 58.25±6.88 | 58.18±7.33 | -0.018 | 0.986 |
| **Laboratory parameters** |  |  |  |  |
| UA(μmol/L,‾*x*±s) | 306.7±96.48 | 334.40±109.14 | 0.538 | 0.599 |
| Cr(μmol/L,‾*x*±s) | 73.89±13.83 | 73.01±20.15 | -0.101 | 0.921 |
| NT-ProBNP(pg/ml,‾*x*±s) | 1429.63±1465.01 | 2363.41±1034.48 | 1.473 | 0.163 |
| HDL-C(mmol/L,‾*x*±s) | 0.98±0.19 | 1.10±0.24 | 1.061 | 0.306 |
| LDL-C(mmol/L,‾*x*±s) | 2.09±0.69 | 2.90±0.89 | 2.025 | 0.062 |
| TG(mmol/L,‾*x*±s) | 1.56±0.51 | 1.52±0.65 | -0.120 | 0.906 |
| TC(mmol/L,‾*x*±s) | 3.55±0.75 | 4.64±1.08 | 2.331 | **0.035** |
| White blood cell count (×10⁹/L, mean±SD) | 6.45±1.64 | 7.25±3.14 | 0.641 | 0.532 |
| Red blood cell count (×10¹²/L, mean±SD) | 4.69±0.56 | 4.41±0.37 | -1.230 | 0.239 |
| Platelet count (×10⁹/L, mean±SD) | 219.88±43.54 | 268.75±95.15 | 1.321 | 0.208 |
| C-reactive protein (mg/L, mean±SD) | 11.79±5.58 | 12.33±5.57 | 0.193 | 0.850 |
| Gamma-glutamyl transferase [U/L, M(P25, P75)] | 21.26(17.25,37.67) | 39.01(29.30,60.17) | 1.995 | **0.046** |
