# Supplementary material for: Whole-transcriptome analysis of Aortic Stenosis reveals dysregulated RNA networks, immune cell infiltration, and NADK2 as a candidate regulator
Source: Hereditas. 2026 Apr 17;163:68. doi: 10.1186/s41065-026-00675-w (PMC13224427; doi:10.1186/s41065-026-00675-w)
Supplement: Supplementary file 3 — Supplementary Material 3. [file 41065_2026_675_MOESM3_ESM.docx]

Supplement Table 2. Statistical results of raw and clean reads.

| Sample | Raw Data | | | Clean Data | | | Clean Reads% |
| --- | --- | --- | --- | --- | --- | --- | --- |
|  | Reads | Q20 | Q30 | Reads | Q20 | Q30 |  |
| CON1 | 55161733 | 95.7% | 91.7% | 49308369 | 97.6% | 94.1% | 89.4% |
| CON2 | 72741055 | 94.8% | 90.0% | 64584552 | 97.0% | 92.8% | 88.8% |
| CON3 | 61335910 | 92.2% | 87.1% | 46399883 | 96.7% | 92.3% | 75.6% |
| CON4 | 68765665 | 92.0% | 87.3% | 51106721 | 96.9% | 92.9% | 74.3% |
| CON5 | 59002501 | 96.1% | 92.6% | 52952874 | 98.0% | 94.9% | 89.7% |
| CON6 | 61528320 | 95.7% | 92.2% | 53498004 | 98.0% | 95.0% | 86.9% |
| CON7 | 71186006 | 94.2% | 90.5% | 57051755 | 97.7% | 94.5% | 80.1% |
| CON8 | 68880964 | 96.9% | 94.0% | 62835479 | 98.4% | 95.8% | 91.2% |
| AS1 | 57673670 | 96.4% | 93.1% | 52054262 | 98.1% | 95.2% | 90.3% |
| AS2 | 48071303 | 97.3% | 94.1% | 46125636 | 98.2% | 95.3% | 96.0% |
| AS3 | 53919681 | 95.3% | 91.4% | 46460767 | 97.6% | 94.2% | 86.2% |
| AS4 | 72301233 | 91.8% | 86.2% | 57316046 | 96.0% | 91.1% | 79.3% |
| AS5 | 59147215 | 94.7% | 90.1% | 53453963 | 97.0% | 93.0% | 90.4% |
| AS6 | 54874611 | 95.9% | 91.8% | 51689072 | 97.4% | 93.8% | 94.2% |
| AS7 | 62820990 | 98.0% | 95.5% | 60737011 | 98.6% | 96.4% | 96.7% |
| AS8 | 57203089 | 96.8% | 93.8% | 52027207 | 98.4% | 95.8% | 91.0% |
