# Supplementary material for: Whole-transcriptome analysis of Aortic Stenosis reveals dysregulated RNA networks, immune cell infiltration, and NADK2 as a candidate regulator
Source: Hereditas. 2026 Apr 17;163:68. doi: 10.1186/s41065-026-00675-w (PMC13224427; doi:10.1186/s41065-026-00675-w)
Supplement: Supplementary file 4 — Supplementary Material 4. [file 41065_2026_675_MOESM4_ESM.docx]

PDP1

BTG2

WDR74

TCTE1

GPR75

FZD10

FN3K

PDE4D

MT1F

ZNF331

OTUB2

OR2B11

MMP19

ZFP36

GBP4

MT1A

PTGS2

IFT20

PLD6

ATP1B3

FNDC11

DDX24

MROH2B

CCNH

ZNF486

ZC3H12A

ITPRIP

RAD54L

TCF7

SLC25A44

TRPM5

JUNB

MAT2A

MTFR2

EIF2AK3

TUBB2B

SLC3A2

LMOD2

RELN

MT1M

KLF9

GJB7

GPM6A

SELL

NR4A3

SLC10A1

IRF1

ZNF655

MYC

OXCT2

NOLC1

SBDS

CCNL1

PRMT9

RNF122

ZFAND5

SLC22A4

SLC41A1

RSPH4A

DCUN1D3

GPR3

IL13

FAM186B

APTX

PHYHIP

MYO1A

OPTC

ARL5B

ID2

C6

IFIT1B

NR4A1

CH25H

MSANTD1

HES1

TAGLN3

CIRBP

EIF1

SCML1

SRMS

SNAI1

ZNF460

RBBP6

LIAS

CD164L2

RIPK4

NCOA7

FASTKD5

ZC3HAV1

TP53RK

USP21

EIF5

SNN

TIPARP

MED20

TUBB2A

TNFAIP3

TSPYL2

NLRP3

EID3

FZD7

OR2H2

NXPH4

TP53INP2

DAPK2

RGPD4

MAEA

POLR2H

KDM6B

RANBP2

NFIL3

EME1

RDH12

WBP11

OVCH2

SFPQ

KCNJ1

BTNL2

RTL1

POLM

JMY

REC8

TDRD12

ZNF346

MT1X

CASQ1

KLF10

MAFF

PLEKHJ1

ZBTB2

CCN1

POLH

MT2A

PPP1R15B

VCP

AZU1

ELL2

CBLN1

AFF4

GCGR

ATP5MF-PTCD1

RAD51

ERICH5

SPDYA

USP2

CSRNP1

WDR55

NAMPT

AP3M2

CTH

GPRC5A

NLRP9

U2AF1

DUSP5

PPM1H

DPYSL4

NOP2

CCR9

TSC22D2

ETV3

MYRF

TRA2B

HSPA9

SNIP1

SLC30A1

FOSL1

HBEGF

ING1

GALR2

NKPD1

SPRED3

LRRC71

ANK2

FIBCD1

GABARAPL1

TXLNG

FUS

SLC19A2

SIRT1

SLC7A5

HBP1

PEX5L

KCNS1

RARA

NMRK2

DDN

EGR3

PXDC1

PSMB9

CPEB2

IL6

FOXK2

CYB5R2

MCL1

TMEM250

ENC1

CAPN10

TNFSF9

WDR45B

PNRC1

IFRD1

ATG101

KPTN

MISP

TMEM88

TTC36

SRFBP1

SWSAP1

ACTL7B

SIK1

PSD

RAB21

BAG3

LY6K

CRISPLD2

CX3CR1

XPNPEP3

MEX3B

WDR4

USP49

CHRM4

OSER1

ID4

FBN2

NSUN5

PANX1

SLC39A12

STXBP6

BCLAF1

POC1B-GALNT4

A1BG

RIPOR3

WBP4

SLC10A5

EIF4A3

ENPP3

SDCBP

PERM1

IL1RAPL1

BTG3

GNA12

LRRC27

WTAP

HSPB3

APBA3

CCL2

YTHDF3

RFXANK

NAPA

MYL4

PHF24
