# Supplementary figures and images for: Whole-transcriptome analysis of Aortic Stenosis reveals dysregulated RNA networks, immune cell infiltration, and NADK2 as a candidate regulator
Source: Hereditas. 2026 Apr 17;163:68. doi: 10.1186/s41065-026-00675-w (PMC13224427; doi:10.1186/s41065-026-00675-w)

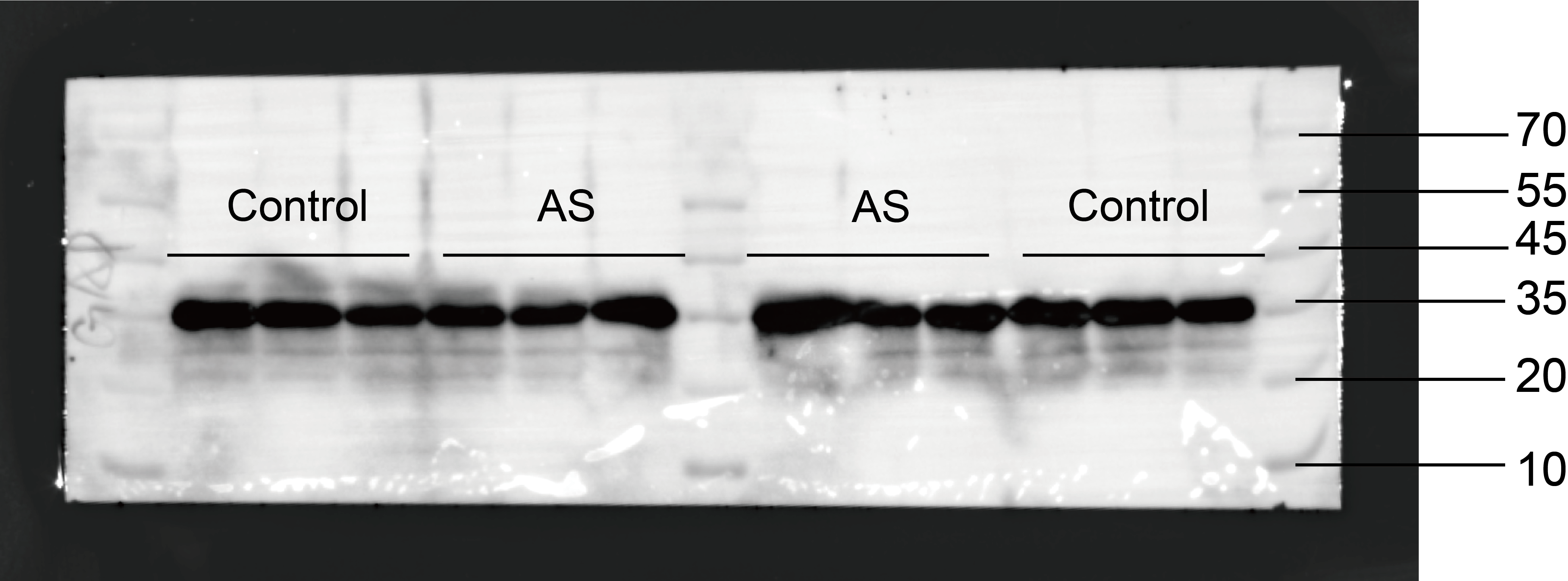

Supplement: Supplementary file 5 — Supplementary Material 5: Supplementary Figure 1. Raw western blot data for GAPDH (human samples). [file 41065_2026_675_MOESM5_ESM.tif]

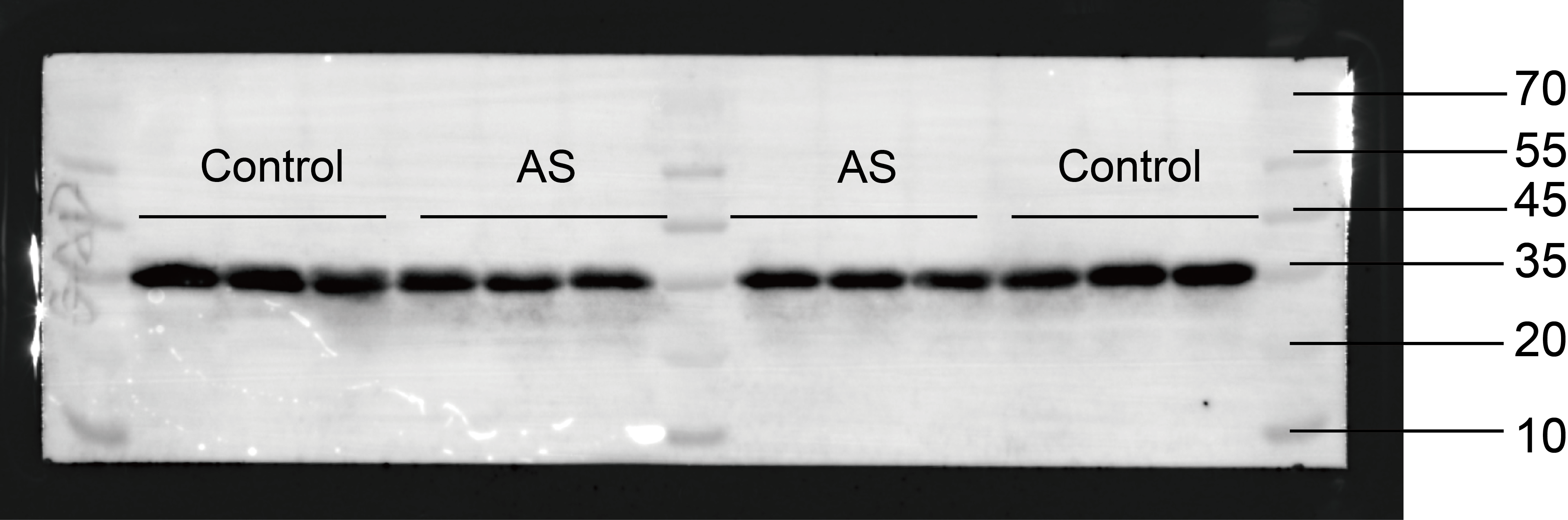

Supplement: Supplementary file 6 — Supplementary Material 6: Supplementary Figure 2. Raw western blot data for GAPDH (rat samples). [file 41065_2026_675_MOESM6_ESM.tif]

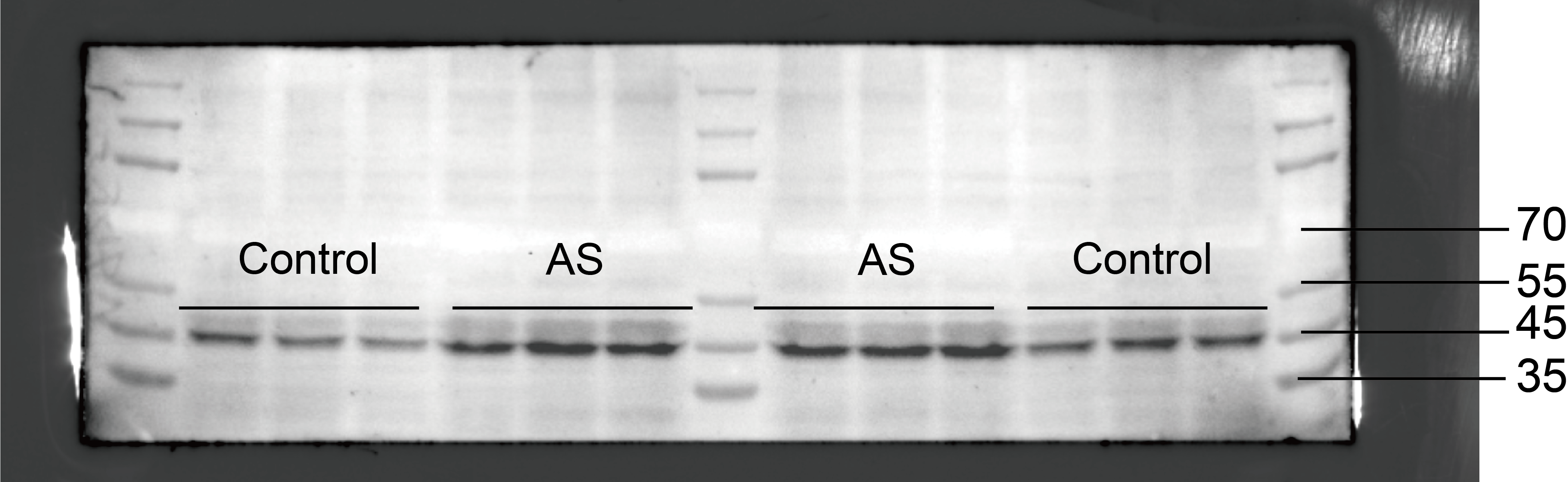

Supplement: Supplementary file 7 — Supplementary Material 7: Supplementary Figure 3. Raw western blot data for NADK2 (rat samples). [file 41065_2026_675_MOESM7_ESM.tif]

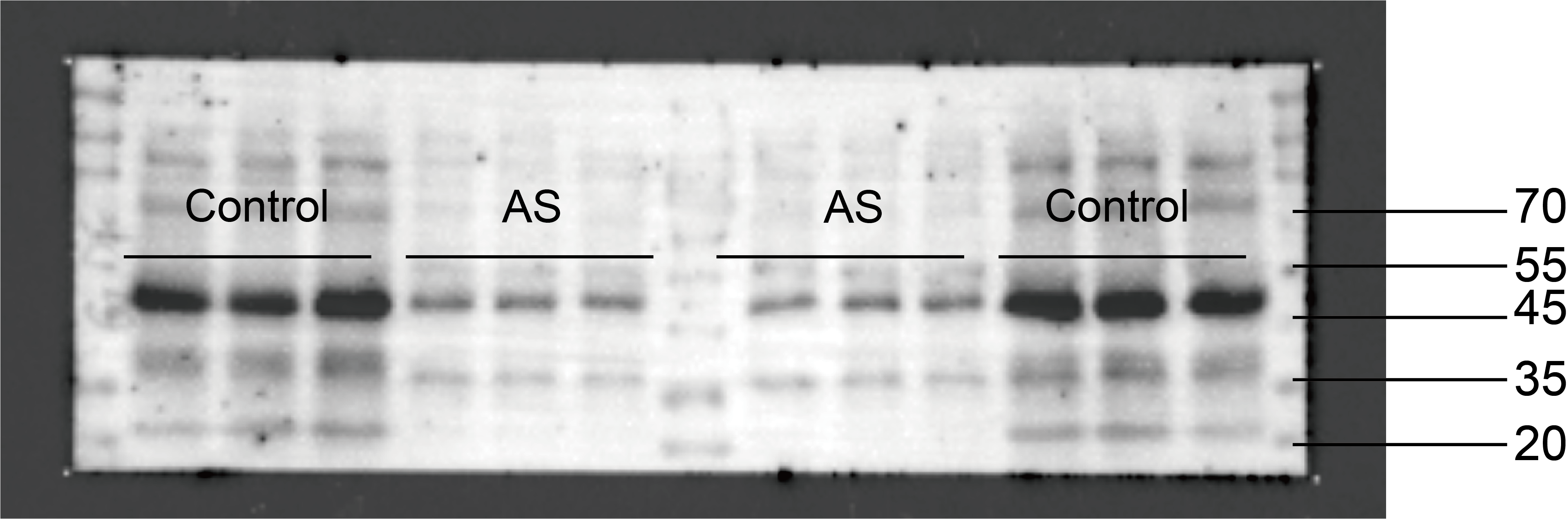

Supplement: Supplementary file 8 — Supplementary Material 8: Supplementary Figure 4. Raw western blot data for NADK2 (human samples). [file 41065_2026_675_MOESM8_ESM.tif]
